# Spatial Variation of the Relationship between PM_**2.5**_ Concentrations and Meteorological Parameters in China

**DOI:** 10.1155/2015/684618

**Published:** 2015-07-29

**Authors:** Gang Lin, Jingying Fu, Dong Jiang, Jianhua Wang, Qiao Wang, Donglin Dong

**Affiliations:** ^1^College of Geoscience and Surveying Engineering, China University of Mining & Technology (Beijing), Ding No. 11 Xueyuan Road, Haidian District, Beijing 100083, China; ^2^Institute of Geographical Sciences and Natural Resources Research, Chinese Academy of Sciences, 11A Datun Road, Chaoyang District, Beijing 100101, China; ^3^University of Chinese Academy of Sciences, Beijing 100101, China; ^4^State Key Laboratory of Simulation and Regulation of Water Cycle in River Basin, Department of Water Resources, China Institute of Hydropower & Water Resources Research, Beijing 100038, China; ^5^Satellite Environmental Application Center, Ministry of Environmental Protection, Beijing 100094, China

## Abstract

Epidemiological studies around the world have reported that fine particulate matter (PM_2.5_) is closely associated with human health. The distribution of PM_2.5_ concentrations is influenced by multiple geographic and socioeconomic factors. Using a remote-sensing-derived PM_2.5_ dataset, this paper explores the relationship between PM_2.5_ concentrations and meteorological parameters and their spatial variance in China for the period 2001–2010. The spatial variations of the relationships between the annual average PM_2.5_, the annual average precipitation (AAP), and the annual average temperature (AAT) were evaluated using the Geographically Weighted Regression (GWR) model. The results indicated that PM_2.5_ had a strong and stable correlation with meteorological parameters. In particular, PM_2.5_ had a negative correlation with precipitation and a positive correlation with temperature. In addition, the relationship between the variables changed over space, and the strong negative correlation between PM_2.5_ and the AAP mainly appeared in the warm temperate semihumid region and northern subtropical humid region in 2001 and 2010, with some localized differences. The strong positive correlation between the PM_2.5_ and the AAT mainly occurred in the mid-temperate semiarid region, the humid, semihumid, and semiarid warm temperate regions, and the northern subtropical humid region in 2001 and 2010.

## 1. Introduction

Many epidemiological studies have shown that long-term exposure to outdoor air pollution could increase the risk of acute and chronic health effects [1]. Fine particles (PM_2.5_, i.e., particles with aerodynamic diameters of less than 2.5 *μ*m), which are the main air pollutants, continue to pose significant threats to health worldwide, especially in developing countries. Chronic exposure to PM_2.5_ has negative effects on human health, including increased morbidity from respiratory problems, cardiovascular problems, and lung cancer [2–4]. Some epidemiological studies have also reported that children have a high susceptibility to respiratory illness when exposed to long-term air pollution due to their developing bodies and immature lungs [5, 6]. In China, the air quality has worsened due to the rapid social and economic development in recent years [7, 8], and more attention has been focused on studies of PM_2.5_. Therefore, long-term PM_2.5_ concentration monitoring and accurate estimations of PM_2.5_ concentrations have become crucial to epidemiological research.

In recent years, the application of satellite remote sensing to air quality research [9, 10], especially the application of aerosol optical depth (AOD) to assessing surface air quality [11], has greatly promoted the estimates of ground-level PM_2.5_ concentrations. Previous studies have established a positive relationship between observed PM_2.5_ concentrations and AOD [12–14]. Kumar et al. [15] and Schäfer et al. [16] examine the relationship between PM_2.5_ and AOD using linear regression. Hu [17] also used Geographically Weighted Regression (GWR) to establish a local relationship between PM_2.5_ and AOD. Many researchers predicted the surface PM_2.5_ concentration by using the relationship between PM_2.5_ and AOD [17, 18].

Local meteorological information can optimize the relationship between AOD and PM_2.5_ [19, 20], and adoption of meteorological information in a model can significantly improve the model's predictability [21]. Paciorek et al. [22] estimated surface PM_2.5_ concentrations with the predictive variables of AOD and meteorological parameters. van Donkelaar et al. [11] used a global chemical transport model with meteorological information to simulate the factors that affect the relationship between AOD and PM_2.5_ and to produce a map of global, annually averaged, remotely sensed PM_2.5_ concentrations for November 2000–October 2002. Based on the methods of Liu et al. [18], van Donkelaar et al. successfully developed a global satellite-based estimate of surface PM_2.5_ using meteorological datasets [23]. However, for meteorological information, previous studies usually focused on the planetary boundary layer and relative humidity as key factors that affect the relationship between PM_2.5_ and AOD [21, 22].

**Figure 1:**
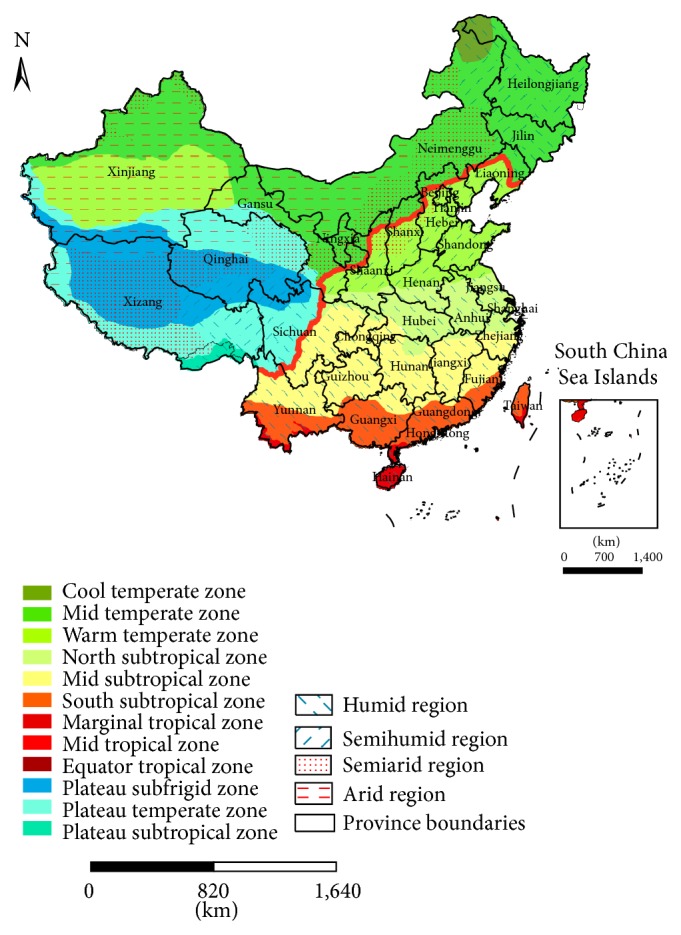
Map of climate zones in China.

In China, the climate exhibits significant differences due to the country's vastness.  Figure 1 shows the climate zones in China [24]. The regions to the right of the red line in Figure 1 are the major economic zones in China, and the PM_2.5_ in these regions that have high populations, high GDP, and large urban areas has become more serious than that in the regions to the left of the line [25]. Many recent studies also note the association between PM_10_ and meteorological factors based on the ordinary least squares (OLS) method at locations in China [26, 27]. However, the relationship between PM_2.5_ and meteorological factors usually exhibits differences in various regions. It is necessary to evaluate the spatial variation of those relationships in China to improve the understanding of air quality and epidemiological studies.

The objective of this paper is to explore the relationship between PM_2.5_ concentrations and meteorological parameters and the associated spatial variance of the relationship in China based on new, long-term raster data. The annual average PM_2.5_ gridded data, annual average precipitation (AAP) gridded data, and annual average temperature (AAT) gridded data for the period 2001–2010 were used in this study. The spatial variation of the correlations between the PM_2.5_ concentrations, the AAP, and the AAT were analyzed using the GWR method.

## 2. Material and Methods

### 2.1. PM_2.5_ Data

The global annual average PM_2.5_ gridded datasets for the period 2001–2010 [28] were downloaded from the website of the Socioeconomic Data and Applications Center (SEDAC). Each data file had a resolution of 0.5° × 0.5°, and all PM_2.5_ concentrations, which were in micrograms per cubic meter (*μ*g/m^3^), were multiplied by 1,000.

Based on the work of van Donkelaar et al. [23], researchers of Battelle Memorial Institute developed a method to estimate annual average surface PM_2.5_ concentrations from AOD. van Donkelaar et al. [23] applied a conversion factor that accounts for the spatiotemporal relationship between PM_2.5_ and AOD to estimate the ground-level concentration of dry 24-hour PM_2.5_. The conversion factor in these datasets was treated as constant during 2001–2010 after minor processing; a relative humidity of 50% was used. The PM_2.5_ concentrations in each grid cell were calculated by multiplying the satellite AOD and the monthly mean conversion factors. Finally, the monthly estimates over each year were averaged to obtain the annual average surface PM_2.5_ concentrations [28]. Compared with the grids produced by van Donkelaar et al. [23], the global estimated PM_2.5_ concentrations from these datasets may have some systematic biases over some regions, such as India and counties in the Sahel, North Africa, and the Arabian peninsula. The bias may be induced by the difficulties of AOD retrievals over sandy surfaces or by the application of the conversion factors [28]. The datasets we used in this paper (China region) are hardly affected by that.

**Figure 2:**
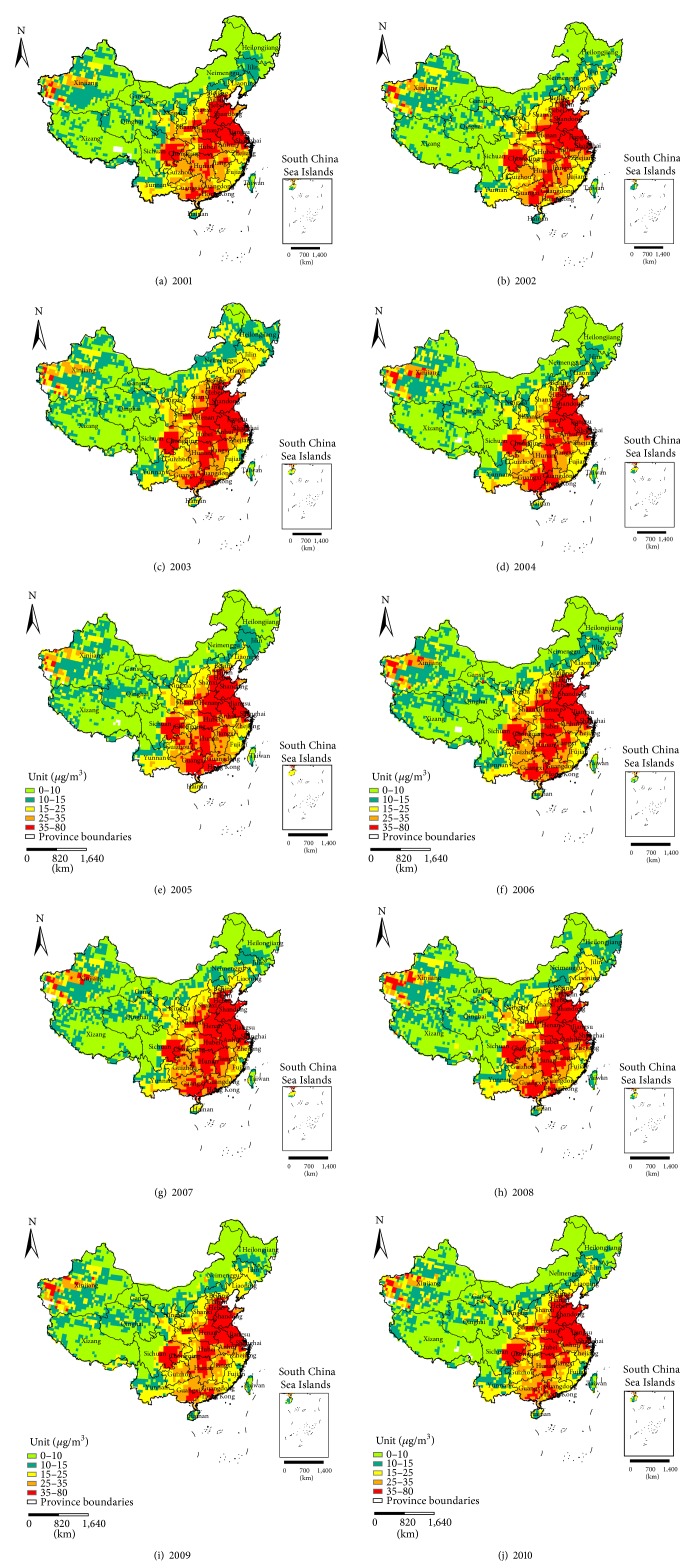
The estimated distribution of PM_2.5_ concentrations in China from (a) 2001 to (j) 2010 (classified according to the WHO air quality guidelines and interim targets).

The China annual average PM_2.5_ gridded datasets for the period 2001–2010 were extracted by ArcGIS software and were transformed to the same coordinate system as the meteorological datasets.  Figure 2 shows the estimated distribution of the PM_2.5_ concentrations in China from 2001 to 2010 according to the WHO air quality guidelines and interim targets [29].

### 2.2. Meteorological Data

The meteorological datasets used in this paper, including the AAP data and AAT data for the period 2001–2010, were provided by the Resources and Environmental Scientific Data Center (RESDC), Chinese Academy of Sciences, and the China Meteorological Administration (CMA). The datasets were in raster format (ArcGIS GRID format) with a spatial resolution of 1 km.

**Figure 3:**
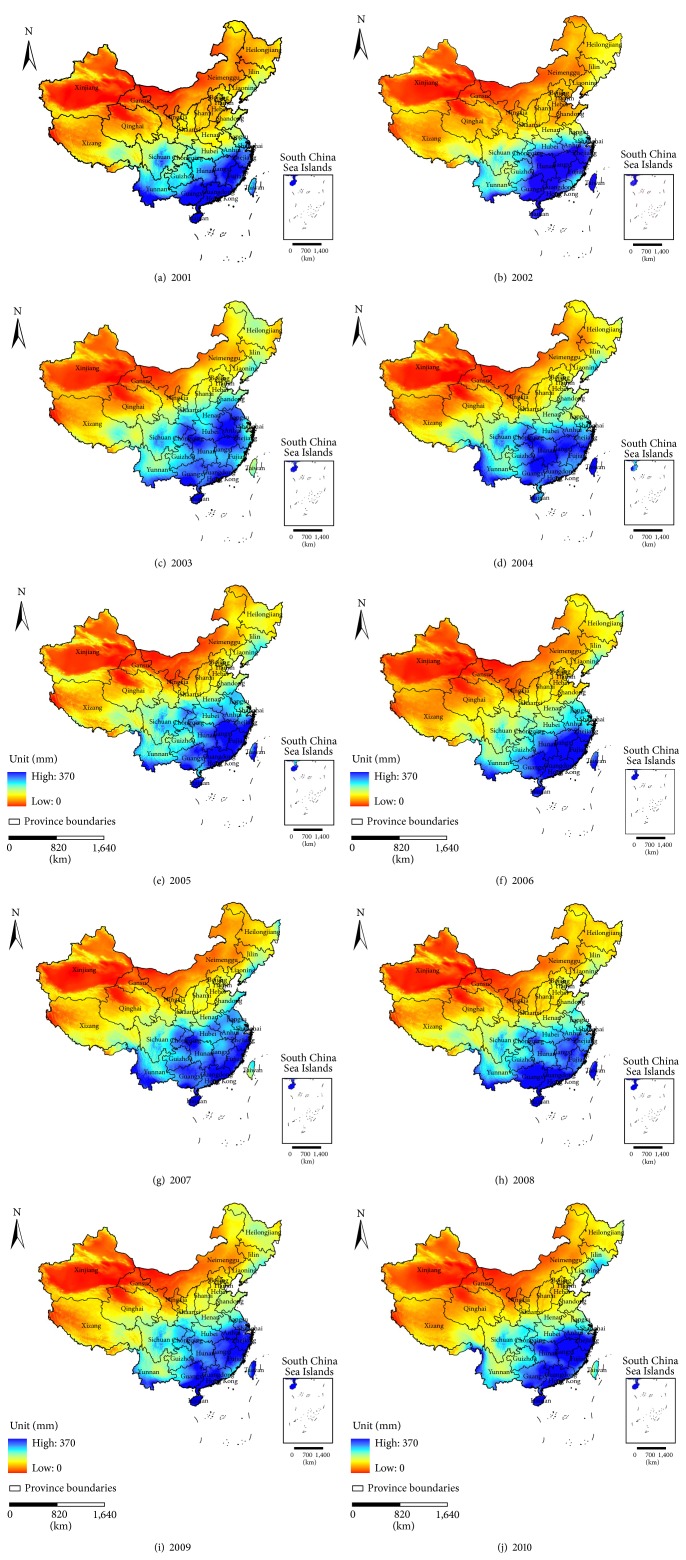
The AAP in China for the period (a) 2001–(j) 2010.

**Figure 4:**
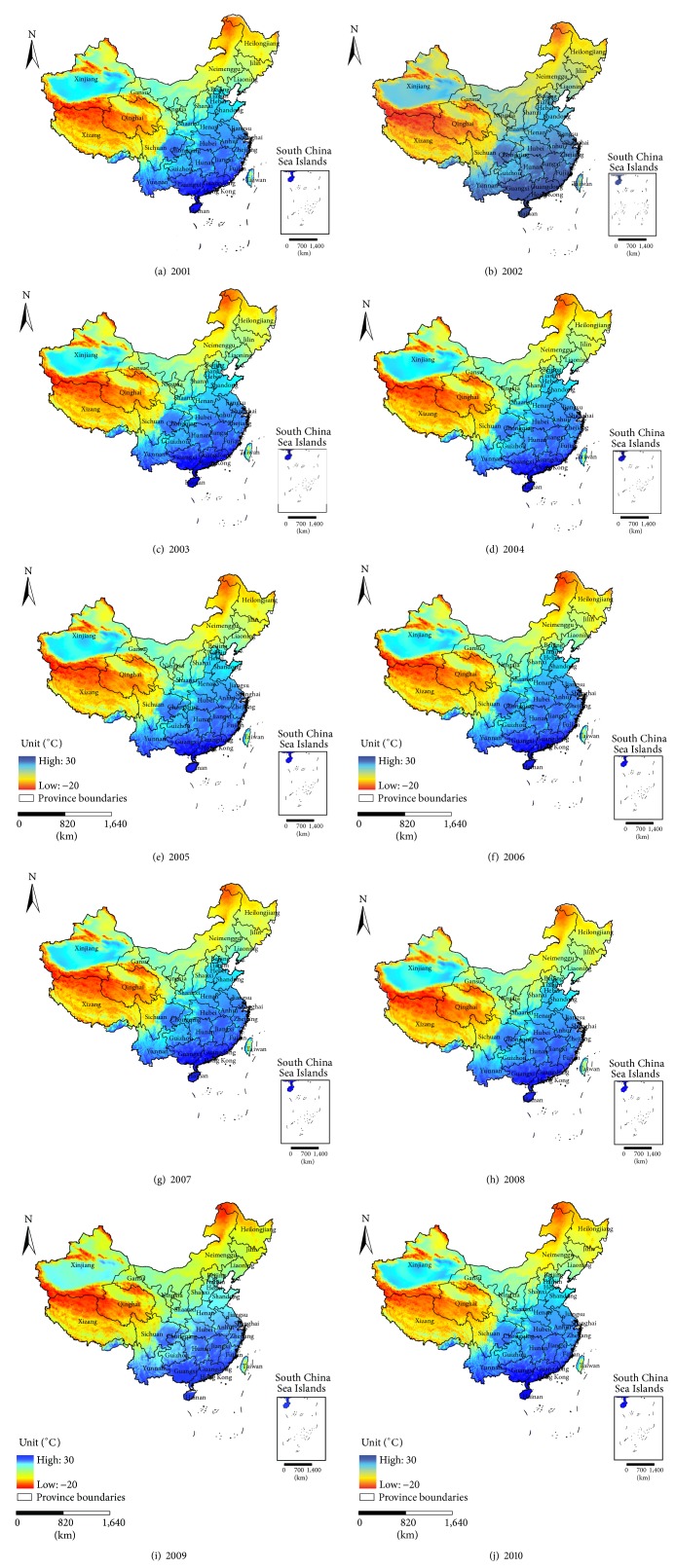
The AAT in China for the period (a) 2001–(j) 2010.

Figures 3 and 4 show the distribution of the AAP and AAT in China for the period 2001–2010.

### 2.3. Methodology

The spatial variance of the relationship between the PM_2.5_ concentrations and meteorological parameters in China was calculated using the following steps.

*Step 1.* Evaluate the relationships between the PM_2.5_ concentrations, AAP, and AAT based on multiple linear regression (MLR) with data of 333 prefectures [30] in China for the period 2001–2010.

*Step 2.* Use the GWR method to explore the spatial variation of the relationship between the PM_2.5_ concentrations, AAP, and AAT for 2001 and 2010.

Based on OLS, Fotheringham et al. [31] developed a method to explore spatial heterogeneity by building a local regression model. The GWR model embeds the spatial locations in regression parameters and considers the local estimates of the parameters. The GWR model can show the influence of independent variables on dependent variables with the locations' changes and the spatial heterogeneity of the relationship between an independent variable and dependent variable [32]. Due to the special characteristics of the GWR, it was adopted in this paper to indicate the spatial variance of the relationship between the PM_2.5_ concentrations, AAP, and AAT.

The global regression model can be expressed as

(1) yi=β0ui,vi+∑kβkui,vixik+εi,i=1,2,…,n,

where (*u*_*i*_, *v*_*i*_) is the spatial coordinate of sample point *i* and *β*_*k*_(*u*_*i*_, *v*_*i*_) is the regression coefficient of sample point *i*. *ε*_*i*_ is the random error of the independent distribution, which is usually assumed to obey a normal distribution. If *β*_*k*_(*u*_1_, *v*_1_) = *β*_*k*_(*u*_2_, *v*_2_) = ⋯*β*_*k*_(*u*_*n*_, *v*_*n*_), then the model will be changed into an OLS model [33].

Bandwidth, which controls the degree of smoothing, is an important parameter for GWR. There are two automatic methods for finding the bandwidth: CV, which minimizes a cross-validation (CV) function, and AIC, which minimizes the Akaike information criterion (AIC) [33]. In this paper, we chose an adaptive kernel by considering the spatial configuration of the features, and we found the bandwidth by minimizing the corrected Akaike information criterion value as it is less biased compared with AIC [34].

## 3. Results

**Figure 5:**
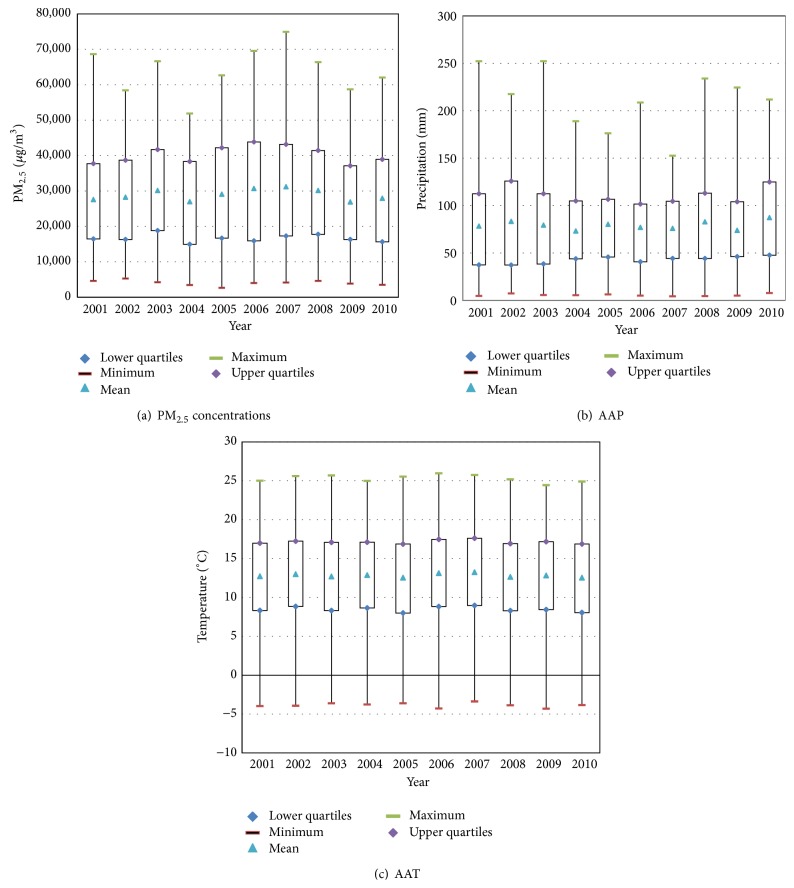
Maps of the summary statistics for the (a) annual average PM_2.5_, (b) AAP, and (c) AAT.

Figure 5 shows the summary statistics of the variables.

Based on the above-mentioned maps, we can see the spatial characteristics of the AAP and AAT and the summary statistics of the PM_2.5_, AAP, and AAT in China for the period 2001–2010. The values of the PM_2.5_ concentrations, AAP, and AAT during the ten years remained at approximately 28 *μ*g/m^3^, 79 mm, and 12.79°C, respectively. Thus, the MLR model was first applied to study the correlation between the PM_2.5_ concentrations and the two meteorological factors. Then, the GWR model was used for evaluating the spatial variation of the relationship between the AAP and AAT in 2001 and 2010.

### 3.1. Correlation between the PM_2.5_ Concentrations and Meteorological Parameters

We developed the MLR model based on the data of 333 prefectures in China using SPSS.  Table 1 shows the results of the model from 2001 to 2010. Based on the results of the MLR, the *R* value reached a maximum of 0.75 in 2001 and a minimum of 0.62 in 2009, with a mean value of 0.688; these results indicate a good and stable correlation between the PM_2.5_ and meteorological parameters in China during 2001–2010. The function for each year also denoted a negative correlation between the PM_2.5_ concentrations and AAP and a positive correlation between the PM_2.5_ concentrations and AAT over the period.

The variance inflation factor (VIF) was used for detecting the existence of the colinearity problem among the indicators via SPSS software. The VIF values of the AAP and AAT were both 2.431 for 2001 and 2.203 for the year 2010. A colinearity problem does not exist among the variables because the VIF values are less than 10 [35].

### 3.2. The Spatial Variation of the Identified Relationship

In the reports of GWR, we obtained the *R* ^2^ and the adjusted *R* ^2^ of the two years, that is, 0.784 and 0.781, respectively, for 2001 and 0.782 and 0.780, respectively, for 2010. The adjusted *R* ^2^ values for both of the years were better than the values obtained in the MLR models. The means of the local *R* ^2^ for 2001 and 2010 were 0.50 and 0.49, respectively. The values for the two years were very similar, which indicates the goodness-of-fit of the models for both of the years.

**Figure 6:**
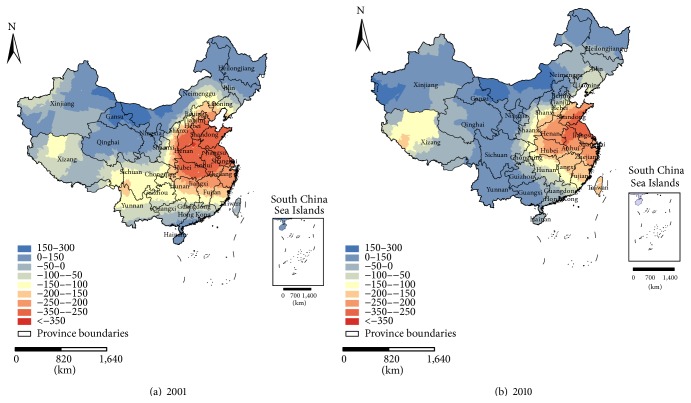
Maps of the local coefficients of the AAP for (a) 2001 and (b) 2010.

**Figure 7:**
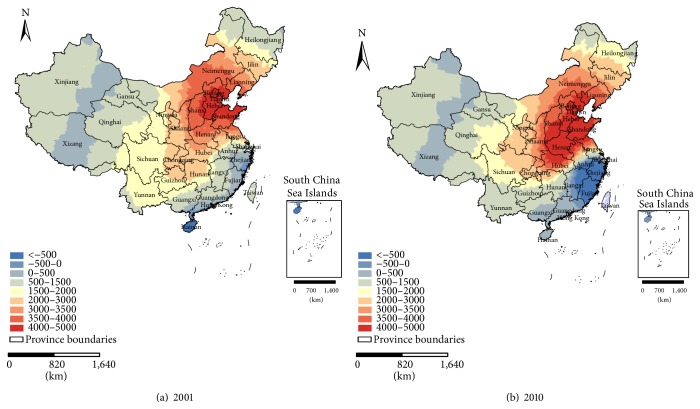
Maps of the local coefficients of the AAT for (a) 2001 and (b) 2010.

Figures 6 and 7 show the spatial variation of the relationship between the PM_2.5_ concentrations and meteorological parameters in 2001 and 2010. Based on the method of Leung et al. [36], in this study, we calculated the *f* = *V*_GWR_/RSS_GWR_ (where *V*_GWR_ is the variance of the coefficients and RSS_GWR_ is the residual sum of squares), which is tested as an *f* statistic to examine the spatial nonstability of the regression coefficients of each variable using an *F*-test. The ratios between the *f* and the corresponding degrees of freedom for each variable in both years were all larger than the critical value at the level of 0.05; thus, the spatial variation of the regression coefficients of the variables was nonstationary.

Figure 6 shows the local coefficients of the AAP for 2001 and 2010. In general, the local coefficients in 2001 indicate that the influence of the AAP on the PM_2.5_ varied considerably over the entire country, with a west-east orientation; approximately 83.8% of the study area exhibits a negative correlation between the PM_2.5_ concentrations and the AAP. The strong correlations with the coefficients below −200 mainly appeared in the warm temperate semihumid region and in the east-central northern subtropical humid region; they rarely emerged in the warm temperate semiarid region or northeast of the mid-subtropical humid region (Figure 1), which includes Beijing, Tianjin, Shanghai, Shandong, Henan, Anhui, Jiangsu, and Zhejiang Provinces and most of Shaanxi, Shanxi, Hebei, Hubei, Hunan, Jiangxi, and Fujian Provinces. In 2010, the overall map is similar to that in 2001, and the influence varies with a west-east orientation. However, the correlations declined in some regions, and only 68.9% of the study area revealed a negative correlation. The strong correlations in 2010 mainly appeared to the southeast of the warm temperate semihumid region and in the east-central area of the northern subtropical humid region; they rarely emerged northeast of the mid-subtropical humid region (Figure 1), which includes Shanghai and Jiangsu Provinces and most of Shandong, Henan, Hubei, Anhui, and Zhejiang Provinces. Compared with 2001, the regions with strong correlations between the PM_2.5_ and AAP were reduced in 2010. Further, in the regions of Yunnan, Guizhou, Sichuan, and Guangxi, the sign of the local coefficient changed by 2010 and may be a consequence of extreme drought and climate change [37, 38].

Figure 7 shows the local coefficients of the AAT for 2001 and 2010. In general, the local coefficients of 2001 indicate that the influence of the AAT on the PM_2.5_ varies considerably over the entire country; approximately 98.8% of the study area reveals a positive correlation between the PM_2.5_ concentrations and the AAT. This finding remarkably agrees with the result obtained from the MLR model. The strong correlations with the coefficients above 3,000 mainly appeared in the mid-temperate semiarid region, the humid, semihumid and semiarid warm temperate regions, and the central area of the northern subtropical humid region; they rarely emerged northeast of the northern subtropical humid region, northeast of the mid-temperate arid region, or south of the humid and semihumid areas of the mid-temperate regions (Figure 1), which include Beijing, Tianjin, Hebei, Shandong, and Shanxi Provinces and most of Liaoning, Neimenggu, Shaanxi, Henan, Hubei, Anhui, and Jiangsu Provinces. Much like the situation in 2001, in 2010, the relationship between the PM_2.5_ and the AAT varied over space; approximately 89.7% of the study area exhibited a positive correlation. The strong correlations mainly appeared in the mid-temperate semiarid region, the humid, semihumid, and semiarid warm temperate regions, and the central and northeastern areas of the northern subtropical humid region; they rarely emerged in the northwestern mid-subtropical humid region, the northeastern mid-temperate arid region, the southern mid-temperate humid region and the southern mid-temperate semihumid region (Figure 1), which include Beijing, Tianjin, Liaoning, Hebei, Shandong, Henan, and Shanxi Provinces and most of Chongqing, Jilin, Neimenggu, Shaanxi, Hubei, Anhui, and Jiangsu Provinces. Although areas with a positive correlation decreased compared to 2001, the regions with a strong relationship increased in 2010, especially in the regions with coefficients above 4,000. Additionally, the sign of the local coefficient changed in a few regions along the southeastern coast in 2010.

## 4. Discussion

**Figure 8:**
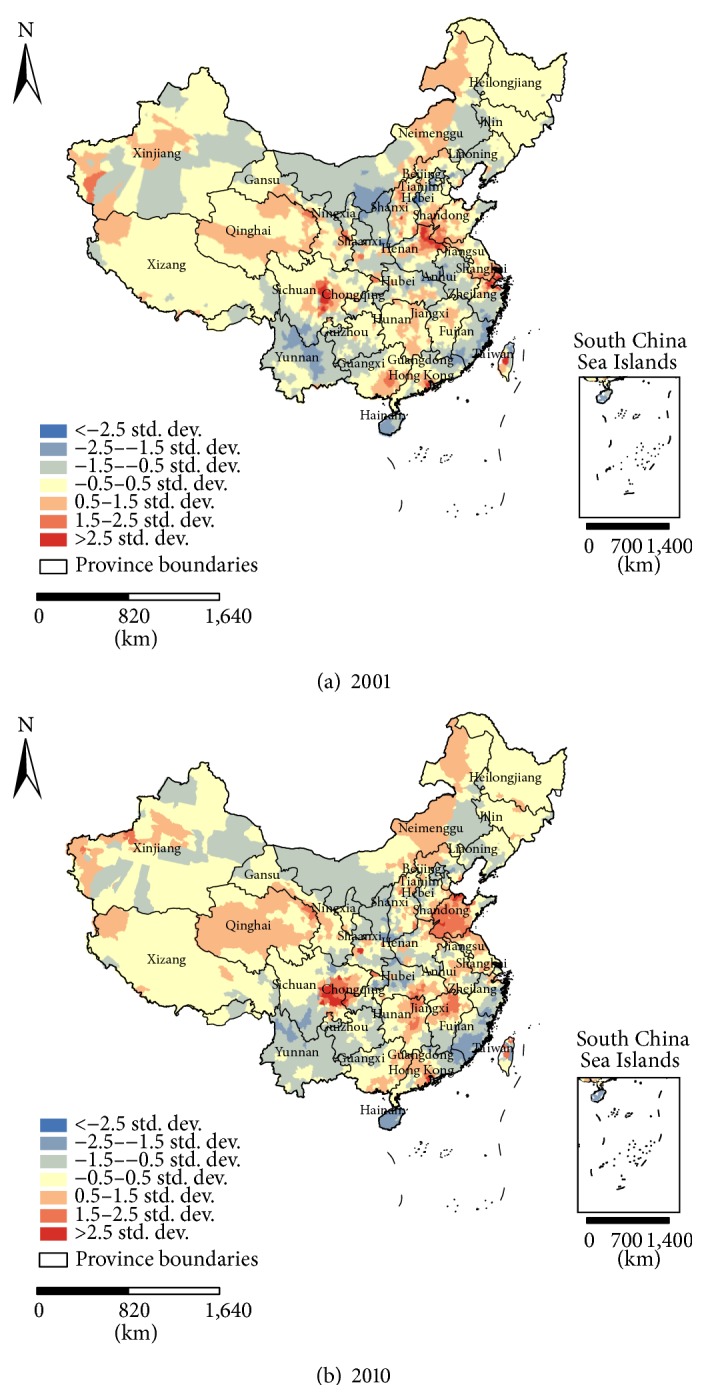
Maps of standardized residuals from the GWR model in China for (a) 2001 and (b) 2010.

It was reported that inclusion of AOD and meteorological fields can significantly improve a model's predictability of surface PM_2.5_ concentrations [19, 21]. Hu et al. [39] built the GWR model to predict the PM_2.5_ concentrations in the Southeast USA, and Tian and Chen [40] also developed a new model to predict the PM_2.5_ concentrations utilizing satellite observations, assimilated meteorological fields, and ground-based meteorological measurements in Pennsylvania, USA. As mentioned above, PM_2.5_ had a strong and stable correlation with meteorological parameters over most of China.  Figure 8 shows the values of the standardized residual (StdResid) for 2001 and 2010 obtained from the GWR model. Regions with StdResid values in the range of −2.5 to 2.5 composed 97.9% (2001) and 96.8% (2010) of the entire country and indicate a better predictive ability for PM_2.5_ concentrations. Although the strength of the correlation between the PM_2.5_ and meteorological parameters varied over space, the parameters are expected to be good predictive variables for estimating PM_2.5_ concentrations when combined with AOD.

The PM_2.5_, precipitation and temperature datasets used in this study were annual averaged values, which may introduce bias when evaluating the relationship between the datasets. There may be a strong seasonal variability in the PM_2.5_ concentration, and the PM_2.5_ concentrations are usually higher during the winter and summer than during the spring and fall [21]. In China, many studies also reported that PM_2.5_ concentrations vary in different seasons [41, 42]. In addition, the precipitation and temperature also exhibit a seasonal variability in China, and high precipitation and temperature are usually associated with the summer. Furthermore, Chen et al. [42] also found that the relationship between PM_2.5_ and meteorological parameters varied in different seasons in Beijing, China. Therefore, the correlation between PM_2.5_ concentrations, precipitation, and temperature would be affected by their seasonal variations. However, the lack of remote sensing datasets of PM_2.5_, precipitation, and temperature in different seasons is the primary obstacle to developing a model to study the relationship between the variables in different seasons. More studies that focus on seasonality are needed if detailed and high-resolution data can be obtained for future work.

## 5. Conclusions

In this study, the relationship between the PM_2.5_ concentrations and meteorological parameters and the spatial variance of the relationship in China during 2001–2010 were evaluated based on newly refined long-term data. The following main conclusions are attained.

(1) The PM_2.5_ had a strong and stable correlation with the meteorological parameters, with a mean *R* value of 0.688 during 2001–2010 in China; thus, a negative correlation existed with the AAP and positive correlation existed with the AAT.
(2) In general, the relationship between the PM_2.5_ and meteorological parameters varied over space. A strong negative correlation between the PM_2.5_ and AAP mainly appeared in the warm temperate semihumid region and the east-central areas of the northern subtropical humid region in 2001 and in the southeastern warm temperate semihumid region and the east-central areas of the northern subtropical humid region in 2010; regions of strong correlations were reduced in 2010 compared with 2001. A strong positive correlation between the PM_2.5_ and the AAT mainly existed in the mid-temperate semiarid region, the humid, semihumid, and semiarid areas of the warm temperate regions, and the central northern subtropical humid region in 2001 and in the mid-temperate semiarid region, the humid, semihumid, and semiarid areas of the warm temperate regions, and the central and northeastern areas of northern subtropical humid region in 2010; the regions of strong correlations increased in 2010 compared to 2001.

This paper advances the study of the relationship between PM_2.5_ and meteorological parameters for the purpose of epidemiological studies. However, the problem is complex and requires more attention in the future.

## Figures and Tables

**Table 1:** The results of the MLR between the PM_2.5_ concentrations and meteorological parameters from 2001 to 2010 (*N* = 333).

Year	Dependent variable (*y*)	Independent variable		*R*	The function^*^
		*x*_1_	*x*_2_		
2001	PM_2.5_ concentrations	AAP	AAT	0.75	*y* = −173.89*x*_1_ + 2448.35*x*_2_ + 10080.71
2002				0.67	*y* = −134.55*x*_1_ + 2156.00*x*_2_ + 11490.86
2003				0.68	*y* = −204.51*x*_1_ + 2582.41*x*_2_ + 13435.09
2004				0.72	*y* = −78.11*x*_1_ + 1879.51*x*_2_ + 8523.51
2005				0.70	*y* = −118.68*x*_1_ + 2228.16*x*_2_ + 10684.55
2006				0.71	*y* = −196.71*x*_1_ + 2718.83*x*_2_ + 10189.49
2007				0.68	*y* = −228.57*x*_1_ + 2747.42*x*_2_ + 12190.28
2008				0.69	*y* = −188.33*x*_1_ + 2649.73*x*_2_ + 12271.67
2009				0.62	*y* = −97.78*x*_1_ + 1750.89*x*_2_ + 11694.93
2010				0.66	*y* = −147.55*x*_1_ + 2257.13*x*_2_ + 12510.72

^*^The functions were valid because they all passed the *F*-test, and all the regression coefficients passed the *t*-test (at the level of 0.05).
